# How do occupational rehabilitation clinicians approach participants on long-term sick leave in order to facilitate return to work? A focus group study

**DOI:** 10.1186/s12913-017-2709-y

**Published:** 2017-11-17

**Authors:** M. Eftedal, A. M. Kvaal, E. Ree, I. Øyeflaten, S. Maeland

**Affiliations:** 1 The National Centre for Occupational Rehabilitation, Rauland, Norway; 2 Uni Research Health, Bergen, Norway; 3 Research Centre for Resilience in Healthcare (SHARE), Faculty of Health Sciences, University of Stavanger, Stavanger, Norway; 4 Department of Occupational Therapy, Physiotherapy and Radiography, Faculty of Health and Social Sciences, Western Norway University of Applied Sciences, Bergen, Norway; 5 Municipality of Vinje, Department of Health and Care Services, Vinje, Norway

**Keywords:** Occupational rehabilitation, Return to work, Focus group, Clinicians, Professionals, Sickness absence, Sick leave

## Abstract

**Background:**

The objective of this study was to explore occupational rehabilitation clinicians’ experiences on how to approach their participants on long-term sick leave in order to facilitate return to work (RTW).

**Methods:**

An exploratory qualitative design was used. Four focus groups were conducted with 29 clinicians working on interdisciplinary inpatient and outpatient occupational rehabilitation teams in Norway. The clinicians shared narratives from clinical practice. Transcripts were analysed, and results were reported by use of systematic text condensation.

**Results:**

The clinicians used several approaches to facilitate RTW among individuals on sick leave. Three themes emerged as especially important in order to succeed: 1) To get a basic understanding of the participant’s life-world through a mapping process; 2) To build a therapeutic alliance through communication characterised by sensitivity to the participants’ needs and emotional concerns; and 3) To initiate processes of change that increase the possibilities for RTW. Four main areas targetable for change were identified, three directed at the individual and one encompassing the participants’ surroundings. These approaches were: a) To increase feelings of confidence and coping; b) To increase the participants’ awareness of their own limits; c) To challenge inefficient and negative attitudes and thoughts related to the sick-role; and d) Close and immediate dialogue with key stakeholders.

**Conclusions:**

To increase the possibilities for RTW among individuals on long-term sick leave, a thorough mapping process and the construction of a therapeutic alliance are seen as crucial elements in approaches by occupational rehabilitation clinicians. By gaining the participants’ trust and identifying their barriers and possibilities for work, the clinicians can target modifiable factors, especially at the individual level, and obstacles for RTW in their individual surroundings. This study elucidates what occupational rehabilitation clinicians do, say and provide to increase their participants’ abilities and possibilities to RTW.

## Background

Long-term sick leave is a major burden for the individual, the family and the workplace, and is costly for society [1]. In Norway, comprehensive occupational rehabilitation programmes may be offered to individuals on sick leave and at risk of dropping out of the labour market due to composite health complaints. Interdisciplinary teams in an outpatient or inpatient setting deliver these programmes. The clinicians provide and co-ordinate services to address the behavioural, functional, medical, physical, psychological, and vocational components of employability and return to work (RTW) for their participants [2]. Musculoskeletal, mental and comorbid disorders are common among those who attend these programmes [3–6]. They also tend to have more complex needs due to the nature of their injury, illness or impairment; length of time they have been off work; home or work circumstances; or other reasons [2]. In the Norwegian context, the prevalence of fatigue, anxiety and depression were found to be high among participants admitted to occupational rehabilitation [7]. In addition, those with sick leave lasting more than one year had a higher prevalence of sleep problems and less social contact with friends, family, co-workers and workplaces. Length of previous sick leave at the time of admittance to occupational rehabilitation was also found to be a strong and independent prognostic factor for prolonged sick leave after occupational rehabilitation in another Norwegian multicentre study [8]. For those on long-term sick leave, RTW might be a challenging and complex process, and many need close follow-up both during and after a rehabilitation programme [4, 9, 10]. It is also argued that the longer anyone is off work, the more difficult occupational rehabilitation becomes [11]. Many of those who attend these programmes do not expect, or are not able to, return to the same job [12], or do not have a job to return to [13]. They may need to find a new job in the labour market in order to reach a sustainable RTW [14]. These processes are time consuming and often beyond the timeframe of the occupational rehabilitation programmes [15]. The quality of the interaction between clinicians and their participants is also vital for rehabilitation to succeed. Good didactic skills and a client-centred attitude are seen as helpful [16]. Many clinicians are inspired by different coaching and dialog techniques, e.g., to enhance their clients’ self-efficacy, empowerment, mental flexibility, learning, motivation for change and goal achievement. They draw on a wide range of social cognitive theories and rehabilitation theories [17–19], including newer behavioural and clinical theories such as acceptance and commitment theory [20], the trans-theoretical stages of change model [21, 22] and motivational interviewing, which draws on self-determination theory and theories of self-regulation [18, 23]. There are several published studies on the use of acceptance and commitment theory in occupational rehabilitation in Norway [5, 24–26].

Both disability and the process of RTW depend on complex and multidimensional factors that should be taken into consideration in the rehabilitation process [27]. There are two particular models that have been used as frameworks by occupational rehabilitation clinicians to understand disability: the International Classification of Functioning, Disability and Health (ICF), which is based on a biopsychosocial perspective [28], and the case-management ecological model of work disability prevention [29]. Even if these models conceptualise the complexity involved and the need for concerted effort and attention by many stakeholders, neither of them fully explain factors leading to disability, work resumption or retention [30].

Regarding evidence-based RTW interventions for individuals with musculoskeletal and mental health disorders, Costa Black presents the results of two synthesis studies [31]. Core components, mentioned for musculoskeletal disorders, include cognitive behavioural approaches, exercise programmes, education to promote self-care and pain management, education/advice about activity and work, protocol-based clinical management and work disability (or ability) assessment. In addition, different components in the interface with workplaces and the stakeholders outside workplaces are found to increase RTW. Many of the same components are described as effective for mental health disorders.

Researchers have also identified several predictive and modifiable factors of RTW that clinicians can screen for and target during occupational rehabilitation at the individual [32, 33], workplace, and system levels [34, 35]. However, because of the complexity and variability of occupational rehabilitation programmes and the multi-causality of sick leave, researchers have not been able to develop validated guidelines for occupational rehabilitation clinicians to follow, as described in several chapters in the “Handbook of Return to Work” [36]. In areas where there are guidelines, the authors also describe implementation problems at the clinics. There are also conflicting results regarding when people on sick leave should be admitted to complex occupational rehabilitation programmes. Early interventions and graded RTW are associated with reductions in sick leave and described as best practice by some researchers [36–38]. It is also argued that many of those admitted to a multidisciplinary intervention programme are admitted too late, increasing the risk of chronicity [39]. However, admission to multidisciplinary interventions too early may also prolong sick leave, and researchers question whether this creates a locking-in effect [40].

Several systematic reviews have found that multi-modal or complex occupational rehabilitation programmes delivered by multidisciplinary or interdisciplinary teams are more effective than single interventions in helping people with musculoskeletal and common mental disorders return to work [10, 31, 41]. Still, there is a need for more knowledge regarding the content of these programmes. Questions raised include how the delivered interventions produce change, how the participants respond to and interact with the interventions, and how contextual factors affect intervention mechanisms and outcomes [42]. Little is known about what clinicians target for change or what they say or provide to address a treatment target during an intervention [43–45]. In order to elaborate the knowledge regarding how clinicians are working and how interventions are delivered to help people return to work, the objective of this study was *to explore how occupational rehabilitation clinicians in Norway approach participants with long-term sick leave to facilitate RTW*.

## Methods

### Design and setting

To elucidate how clinicians work with individuals on long-term sick leave, we chose a qualitative study design with a pragmatic approach. This approach allows for the study of authentic themes raised by clinicians, independent from any predetermined theoretical framework set by the researchers [46]. A focus-group design was chosen, as it is a social context that enables participants to interact with each other by sharing and reflecting on each other’s experiences and ways of working during the session [46, 47]. Dialogue in groups also stimulates the clinicians’ memory of similar or different patient-stories, giving more rich data. We report this study following the consolidated criteria for reporting qualitative research (COREQ) by Tong and colleagues [48] and O’Brian and colleagues [49].

The research group covers several disciplinary backgrounds, including physiotherapy (Silje Mæland, PhD; Irene Øyeflaten, PhD; Monica Eftedal, PhD), nursing (Astrid M. Kvaal, MSc), psychology (Eline Ree, PhD) and sociology (Monica Eftedal, PhD). Three of the authors have clinical experience working within occupational rehabilitation in Norway.

### The Norwegian insurance and benefit system

Residents in Norway are covered by the National Insurance Act and have membership in the National Insurance Scheme, which sets the criteria for benefits and services from the Norwegian Labour and Welfare Administration (NAV). During the first year of sick leave, employees in Norway are entitled to sickness benefits equal to their income (upper limit 6G; 1 G equals NOK 90,068 (2015)), or loss of income in graded sick leave. The work assessment allowance has an upper time limit of 4 years and is assigned to individuals who are undergoing medical treatment or rehabilitation, or who may return to work after vocational rehabilitation.

### Selection of participants and procedure

We recruited clinicians working in occupational rehabilitation clinics when they registered for participation in an annual national network meeting. Twenty-nine (57%) clinicians agreed to participate. They worked as team leaders, physicians, work consultants, occupational therapists, physiotherapists or coaches. Eighty-three per cent were women, and the mean age was 46 years (range: 29–65 years). The study population represented 18 of 25 different inpatient or outpatient clinics that had joined an occupational rehabilitation network in Norway. Four participants came from four different outpatient clinics, and 25 participants came from 14 different inpatient clinics. All participants worked on interdisciplinary teams and had clinical experience with individuals on long-term sick leave. The mean length of clinical experience was 6 years (range: 1–17 years). All institutions deliver comprehensive interdisciplinary occupational rehabilitation programmes in the specialised health care setting. The interventions encompass some form of individual- or group-based cognitive behavioural approach, physical exercise and education, work-related components and dialogue with key stakeholders.

### Data collection

The informants were divided into the following four focus groups: 1) four team leaders and four physicians; 2) eight work consultants; 3) six clinicians with health backgrounds working mainly as coaches or counsellors; and 4) seven physiotherapists. We were aiming for homogeneity regarding profession or role so that they could more easily identify with each other’s experiences across institutions. Owing to the differences in numbers and constrictions regarding resources and timespan, some groups had a mix of professionals. The focus groups were conducted simultaneously. In each group, a moderator facilitated and guided the discussion based on an interview guide, and a co-moderator took notes and evaluated the atmosphere and interaction during the session. The informants were invited to share their experiences with how they approach participants on long-term sick leave, the evaluations they made and their choice of interventions. The interviews, lasting for 1.5 h, were audio recorded, encrypted and transcribed verbatim.

### Data analysis

The transcripts from the interviews were analysed using qualitative content analysis with systematic text condensation, which is a descriptive, cross-case analysis strategy [47].

All members of the research group read all transcripts to obtain an overall impression. After negotiation, the following three main higher-ordered themes were agreed upon as important for the study question: “Basic mapping of the participants’ life world”; “Therapeutic alliance”; and “To facilitate processes of change”. Next, all text were imported in QSR NVivo 10, and meaning units representing main themes were identified and coded (ME). Then, all text was systematically read line by line in an inductive approach to identify subthemes (AMK and ME). In the initial stage of coding, the clinicians’ use of concepts, describing the theme they were talking about, was used as codes (in vivo nodes). New insight from the data was used to rename and organise codes. For example, the clinicians’ use of concepts such as “trust” and “alliance” was categorised as “therapeutic alliance”. The clinicians’ descriptions of how they tried to gain their participants trust were coded by their own words, such as honesty, listening, respect, etc., and sorted under the subgroup “approach”. In the final step, the content of each code group was summarised to generalised descriptions by use of systematic text condensation and combined with illustrative quotations (AMK, ER and ME).

## Results

### The clinicians do a thorough interdisciplinary, basic mapping of the participants’ life-world to enable targeted interventions

According to the clinicians, diagnosis and duration of sick leave are not sufficient to decide how to approach individual participants. To succeed, they needed a thorough mapping process to adapt the interventions to individual needs. The clinicians described many of those who attend occupational rehabilitation programmes as “*trapped in a vicious circle*” marked by many concerns and reduced coping abilities. The way the participants presented themselves to the clinicians was also described as complex. According to the clinicians, lack of coping at work, conflicts, great care burdens and concerns related to health, family, divorce and economic issues are common. As time goes by, many lose belief in their own abilities and opportunities to return to work, becoming more and more entrenched in their own thought patterns. To help the participants, the clinicians described to form a comprehensive picture of the participants’ situations, which included their barriers, resources and possibilities for RTW. The clinicians tried to assess whether the participants were struggling with physical and/or mental complaints, and they also looked for connections between mindsets, bodily reactions and symptoms. The clinicians especially emphasised their interest in how the participants thought about their own situations and functions, their self-understanding and their attitudes and expectations regarding RTW. One of the clinicians described this process by use of a metaphor:

*“Often, I find that the participants are like an onion. They present the onion as a whole and then perhaps by asking the right questions, layer by layer is peeled off.” (Group 3: nurse, 2 years in occupational rehabilitation)*

The clinicians stated that an ICF perspective is the foundation of their mapping, using questionnaires, examination, dialogue, and observations to evaluate the participants’ functions and participation in work and social life. Visual tools were described as especially important in the mapping process; that is, according to the clinicians, tools that increase both the participants’ and the clinicians’ understandings of barriers and resources, and are therefore useful in dialogue with the participants. The participants are given a simple exercise to reflect upon their situation as a whole, often by colouring a drawing of a shoe or a clock. The shoe is divided into different fields representing work, circadian rhythms, finances, children, family and self, visualising their resources and challenges. The clock visualises how they spend their day and what gives or drains them of energy. A clinician noted that:

*“When they get that kind of visual image, their reactions are often a bit like: ‘ooh, is this how my life really looks’ and they start to tell (their story).” (Group 3: nurse, 2 years in occupational rehabilitation)*

The clinicians stressed the importance of their interdisciplinary team approach as it gives them the possibility to gain a more comprehensive picture of their participants’ challenges and resources. According to their experiences, the participants often tell different stories to their physician, physiotherapist or other clinicians in line with their own expectations of what concerns those clinicians. Furthermore, as a team, they have the opportunity to observe and discuss how the participants function both physically and socially in different settings during the intervention. Additional information collected from key stakeholders, such as the referring general practitioners (GPs), employers and NAV, are described as important in their mapping process. These stakeholders might also bring perspectives that differ from those of the participants, and they can give an overview of previous efforts and further action plans for RTW.

### The clinicians build a therapeutic alliance to initiate individual change processes among their participants

A relationship based on mutual understanding and trust was considered a prerequisite by the clinicians to be able to initiate individual change processes among their participants. They described several key elements needed to establish a therapeutic alliance, such as creating a positive atmosphere characterised by openness, honesty and respect, recognition of the individual participants and their needs, as well as having sufficient time during consultations. In addition, the clinicians emphasised the importance of being perceived as competent, interested and supportive by their participants in order for them to trust the clinicians and summon the courage to tell their stories. They kept in mind, or asked directly, what they could do to become a good resource for the participants.

Several clinicians described episodes where they did not manage to establish the therapeutic alliance and trust they needed. Their explanations of why this occurred were multifaceted. As they described, the participants could experience the clinicians as less competent or experienced, or not really interested in their story. The participants could also have had unrealistic expectations regarding what the clinicians would involve themselves in. The clinicians also mentioned reactions from their participants that made them understand that they had not been sensitive enough to the participants’ emotional concerns, such as *“moving too fast*” or using *“wrong words*”. According to the clinicians, several participants bring negative experiences with them, e.g., not being listened to or being met with suspicion when presenting their problems to clinicians and caseworkers. If they experience occupational rehabilitation clinicians in the same way, this may be interpreted as a new violation by the participants, and thus hinder the clinician’s possibilities to initiate any change processes. When the clinicians sensed such difficulties, they tried to demonstrate a more trusting and supportive relationship by their communicative responses, as noted by one of the clinicians:

*“You say you have pain and I believe that. You do not have to prove to me how much pain you have.” (Group 1: team manager, 7 years in occupational rehabilitation)*

The clinicians also referred to some groups of participants that were especially challenging to establish a trusting relationship or facilitate individual change processes with. In the clinicians’ experiences, these participants typically have an unresolved health situation, an ongoing insurance claim or have been referred to the clinics by NAV after long-term sick leave. Those with an unresolved health situation were described as reluctant to challenge themselves because of fear of getting worse. The others were experienced as being more interested in showing what they could not manage rather than actively participating in the rehabilitation process. They were described as being more suspicious toward the clinicians’ roles and more anxious of revealing too much personal information in the case in which their disclosure would negatively affect decisions regarding sickness benefits, insurance or disability pension.

*“In a way, we are (perceived as) NAV’s extended arm, and then their ‘spikes’ are out.” (Group 3, work consultant, 2.5 years in occupational rehabilitation)*

### The clinicians initiate processes of change that increase the participants’ abilities and possibilities for RTW

Based on all of the information the team gathers through the mapping process, they discuss the goal of the rehabilitation process for each participant in team meetings and agree upon the best way to approach the participants’ challenges during the programme. In addition, the participants are involved in decisions regarding their rehabilitation process and goal setting, defining where they are, where they want to be and how to get there. Also, as the clinicians stated, they try in several ways to *“grind down the threshold to working life”* for their participants by identifying barriers, resources and possibilities for RTW, working with individual change processes and initiating dialogue and co-ordination of activities with key stakeholders in order to increase the possibility of RTW.

The clinicians described four main approaches to facilitate changes: three directed at the individual and one encompassing the participants’ surroundings. These approaches were: a) To increase feelings of confidence and coping; b) To increase the participants’ awareness of their own limits; c) To challenge inefficient and negative attitudes and thoughts related to the sick-role; and d) Close and immediate dialogue with key stakeholders.

#### Increasing feelings of confidence and coping are essential for the rehabilitation process

According to the clinicians, many of their participants had been sedentary for a long time when they were admitted to occupational rehabilitation. In their opinion, many of them had developed behaviour characterized by fear avoidance and pain catastrophizing because they interpreted their bodily reactions and symptoms as harmful. To increase the participants’ feelings of confidence and coping, the clinicians noted that they used a lot of activity to facilitate coping experiences. The rehabilitation process is often introduced with a combination of light physical activity and education on different topics, such as anatomy, physiology and normal bodily reactions. As the clinicians noted, they tried to give the participants both experience and knowledge to understand that *“it is not dangerous to bend forward”*. Gradually, the participants are challenged physically, for example, in wall climbing, or subjected to interval sessions where they get to experience that a rapid heartbeat, rapid breathing or a taste of blood during intense exercise is not harmful. Furthermore, the clinicians also mentioned that they used mobility exercises, relaxation techniques and mindfulness to increase the participants’ body awareness. Pain drawings, where the participants mark the areas that hurt, are also used. This is a visual tool that is used to help participants rediscover the healthy parts of their body and what works despite the pain. As the clinicians described, they tried to normalise the views of the participants in relation to function, pain and pain behaviour. Eventually, the participants’ goals might change from being completely free of pain to being able to stay at work in spite of being in pain. One of the clinicians described their approach as follows:

*“It is about making things more harmless (...) recognise their symptoms, but at the same time giving them the faith that they don't have to limit themselves in the same way as they might have done until now. Don't let it be disabling even if they have their ailments.” (Group 4: physiotherapist, 5.5 years in occupational rehabilitation)*

Clinicians found the complexity of participants who had been out of employment for a long time especially challenging. According to them, these participants typically find RTW so scary that disability pension is seen as their only solution. In the clinicians’ opinions, the employers are also more often reluctant to hire them. The clinicians described that their strategy in such cases was not to reinforce the idea about disability pension, but rather to bring out positive memories associated with being at work. They may ask the participants to describe situations where they experienced job satisfaction, memories of tasks they liked to do, the importance of belongingness, good colleagues and being part of the social community. Then, they go on encouraging them to reflect upon what a future life as a disability pensioner would be by asking questions such as *“What would you achieve?”* and *“What would you lose?”*. In addition, some participants may need a lot of practical support to overcome their fear of work and think more positively about their capabilities. One of the clinicians gave an example of how they supported a woman who had been out of work for 11 years in order to gain suitable employment tailored to her needs:

*“We agreed that she should try ‘assistance in work accommodations’. She wanted to try this in a grocery store that had recently opened. We contacted them (the owners), but they would not take on anyone for work training. OK, we said, let us apply then. She applied for that position and we did a lot of interview training (…). Eventually, she made the interview and got the job. This was about 2–3 years ago. Now she has moved from a 50% to a 100% position” (Group 2: work consultant, 10 years in occupational rehabilitation/follow-up of individuals on long-term sick leave)*

#### Some participants need to increase their awareness of own limits to manage work

According to the clinicians, exhaustion because of a demanding job and life situations is typical among many rehabilitation participants. These participants are described as perfectionists never satisfied with their accomplishments combined with a lack of awareness regarding their own limits. A frequent description of work orientation among these participants given by clinicians is that many of them are too involved in their job and career, and have “*hit the wall*”. An important goal for the clinicians, they said, is to make the participants reflect upon where “*the shoe pinches*”, what “*drains them of energy*” and what *“drives them*”, initially through visualisation tools. Next, the clinicians pose questions that help the participants set realistic goals for RTW, realise that they need to slow down and understand that it is actually “*not possible to deliver 150% at work 100% of the time*”. In addition, clinicians try to help them understand that being out of full- or part-time work is acceptable.

Another group of exhausted participants without reasonable limits for themselves, as stated by the clinicians, involves those who are always available to fulfil the needs of others. They let others “*be in the driving seat*” and “*control their lives*”, they said. A typical participant in this category, according the clinicians, is a mum. The clinicians described how they could increase their awareness by asking questions regarding the importance of the tasks they were doing; the possibility of allocating responsibility and prioritising their own needs.

#### To challenge inefficient and negative attitudes and thoughts related to the sick-role

According to the clinicians, some participants think the last day on sick leave is the time to return to work. Others believe they need to be absent from work as long as they have symptoms. In both cases, the clinicians explained that they teach the participants about their rights and obligations when they are on sick leave. They also said that they have a dialogue with the participants to help them sort out the obstacles to their job. In addition, they try to identify whether anything can be done to help their participants achieve earlier RTW. In this process, the clinicians noted, they often ask the participants to reflect upon whether staying at home or being at work is the best option for them.

According to the clinicians, a common problem when people are on sick leave is that many become concerned with the expectations of others regarding the appropriate actions to take. As a result, they behave in ways that are not beneficial for their health. That is, they stop exercising and start avoiding social contact and participation in pleasurable activities such as going to cafés, the cinema and so on. The clinicians also spoke about participants who walk or travel long distances from home to avoid occasional contact with their supervisors and colleagues when they go out for exercise or shopping. They are afraid of what their supervisor or colleagues might think or ask if they meet them. The clinicians’ approach when they identified these patterns, they noted, is to make the participants reflect on restrictions they put on themselves, and to encourage positive thoughts and attitudes. One of the clinicians illustrated this by telling a story of a female cab driver who did not dare step out of her house because she feared what others would think if they saw her outside while she was on sick leave:

*“I had to ask her: “How do you see others that are on sick leave?” (…) I confronted her with the fact that she has two set of standards – one for others and one for herself.”(Group 1: physician, 20 years in occupational rehabilitation)*

#### Co-ordination of activities and dialogue with key stakeholders

Close collaboration with the employer, the general practitioner and the local NAV-consultant, or other services in the municipalities, were emphasised as vital for a successful RTW by the clinicians. They described the dialogue with the employer before and during the participants’ stay at the clinics as essential. As they noted, employers can give their views on how the employee manages her/his job, and tell if there are possibilities for modified work or work accommodations when needed. Examples of accommodations at the workplace that their participants often needed were easier physical labour, other types of tasks or reduced working hours. They often experienced that the dialogue with the employer revealed whether the participant could return to the same job after completion of the rehabilitation programme, or whether she/he should change jobs. According to the clinicians, a dialogue with the employer often resolves important issues and is usually worth the effort. However, many of the clinicians experienced the restricted time limit of their occupational rehabilitation programmes as a problem. With limited possibilities to follow up after the programme, they must rely on other stakeholders’ efforts, but as they stated, they usually know very little of this process because it is outside of their responsibility.

The clinicians described that many of those who attend occupational rehabilitation have psychological challenges connected to relational problems and conflicts at work. Sometimes, these conflicts are too entrenched to resolve, and assistance in finding a new job was the only solution experienced by the clinicians. However, they noted several examples of being able to help participants return to the same job by establishing a dialogue with the workplace and teaching the participants principles of good communication. Clinicians expressed that they tried to increase the participants’ own awareness of how they communicate and give them knowledge and skills regarding how their communication could be improved. Some clinicians said that they discuss or attempt approaches that the participants can use in challenging situations. One clinician illustrated their approach by sharing a story of a woman who had been out of work for 2.5 years because of a conflict with her supervisor. She had no faith in her possibilities of a RTW:

*“First, we worked intensely at the clinic with her thoughts and what was wise to say and do (…) and at the end of her stay, we visited the workplace. The visit was very positive. The HR consultant took hold and was incredibly benevolent in relation to facilitation (…) the immediate supervisor had also chosen a humble approach. We agreed to start very cautiously. After six months, she stepped up. It went very well (…) and she was regularly followed up by her supervisor (…), the personnel leader and some of us from the rehabilitation centre were always present at meetings (…) and she had in a way regained the belief that this would work well.” (Group 2: work consultant, 9 years in occupational rehabilitation)*

## Discussion

The aim of this qualitative study was to explore how Norwegian rehabilitation professionals approach participants with long-term sick leave in order to facilitate RTW using narratives from clinical practice. Three themes emerged as especially important in the occupational rehabilitation process: 1) To get a basic understanding of the participants’ life-worlds through a mapping process; 2) To build a therapeutic alliance through communication characterised by sensitivity to the participants’ needs and emotional concerns; and 3) To initiate processes of change that increase the participants’ possibilities for RTW. Four main areas targeted for change were identified, three directed at the individual and one encompassing the participants’ surroundings: a) To increase feelings of confidence and coping; b) To increase the participants’ awareness of their own limits; c) To challenge inefficient and negative attitudes and thoughts related to the sick-role; and d) Close and immediate dialogue with key stakeholders.

The interventions the clinicians described as approaches to enhance RTW among their participants on long-term sick leave were in line with what Costa-Black (2013) referred to as the core components of evidence-based RTW interventions for individuals with musculoskeletal and common mental disorders [31, Table 26.1]. Costa-Black classified the core intervention components in three corresponding interfaces of interaction: interface with workers; interface with workplaces; and interface with stakeholders. In the interface with workers, the clinicians in the present study described that they used cognitive behavioural approaches, in either one-to-one or group sessions. The participants’ expectations, beliefs, self-efficacy, personal control, attention to pain stimuli, coping, inefficient and negative attitudes and thoughts were addressed. To increase their participants’ work ability, they used exercise programmes, both with and without job specificity. Education were used to increase their participants’ understanding of their own problems, and to promote self-care and pain management. Education and advice about activity and work were also used to help their participants set goals for RTW and find appropriate levels of activity at both home and work. Disability factors that may hinder or promote RTW were assessed through questionnaires, interviews and other tools. Visual tools were mentioned in this study as means especially important for supporting dialogue with the participants, and increasing mutual insight and understanding. This was also described as important in a recent Norwegian trial that evaluated a new tool for occupational rehabilitation clinicians [6].

The clinicians in our study used what is labelled as a patient- or client-centred approach in communication with their participants [50, 51]. As the clinicians demonstrated, they strived for communication characterised by sensitivity to the participants’ needs and emotional concerns, empathic and compassionate care, trust and mutual understanding. They listened to what the participants had to say and asked open-ended questions with an encouraging and supportive attitude in order to help them reflect on their situations. They also identified and acknowledged their resources and possibilities for RTW, and assisted them in their goal setting and process toward RTW. The clinicians’ goal, as they described it, was to empower their participants, increase their beliefs in their own abilities and motivate them to make changes that increased their probability for RTW. Creating positive encounters was also described as a goal in order to increase the therapeutic alliance, which was seen as a prerequisite by the clinicians for the participants to adhere to the rehabilitation programme, and for the clinicians’ possibilities to initiate individual change processes. This approach is supported by research. A systematic review found that patient-centred communication is associated with positive therapeutic alliance [50]. The approach is also seen as vital to achieve consensus and adherence to the goals of treatment and to initiate individual change processes [52–55]. An empathetic attitude from the clinicians may also play a beneficial role in healing beyond the mere effect of the therapeutic alliance on the patients [56]. Positive healthcare encounters give the participants feelings of ownership, and of being believed, confirmed and listened to, in addition to opportunities for increased self-understanding [57–60].

In the initial mapping process, the immediate goal of the clinicians was to identify modifiable barriers for RTW at the individual and contextual level, which they were able to target with their interventions. As Marois and colleagues (2009) found, screening for predictive factors and obstacles at the time of admission to the programme appears to promote RTW [61]. The clinicians in this study were especially focused on individual factors such as negative attitudes and beliefs (negative expectations for resuming work, self-efficacy and motivation for RTW), and behaviour (fear avoidance, lack of coping and decreased physical activity), as supported in other research [32, 33, 62]. In addition, the occupational rehabilitation clinicians’ also paid attention to the participants’ abilities and needs in order to limit their involvement in work and private lives and balance demands in a sustainable way. This is in accordance with challenges and approaches identified in research on patients with fibromyalgia and stress-related disorders [63, 64].

Interface with stakeholders encompasses administrative provisions, communication between stakeholders, team-based approaches and RTW co-ordination or case management, according to Costa-Black [31]. The clinicians in our study viewed the interdisciplinary team-based approach as a major advantage. As they noted, this approach allowed them to discuss cases with each other from different professional perspectives and share information from observations of, and dialogue with, the participants throughout the occupational rehabilitation programme. According to the clinicians, this increased their possibilities to understand the participants’ problems, establish joint treatment goals and strategies, and adjust and individualise their approaches. The significance of interdisciplinary teamwork for successful multimodal work-related rehabilitation is also underlined in previous research [29, 65]. In our study, the clinicians described the team-based approach as an especially advantageous in cases where the participants were not motivated for RTW, not interested in co-operation or lacked trust in the clinician. As they explained, different clinicians use different approaches and have different knowledge; this increases their possibilities to gain the trust and alliance they need in order to help people RTW. Some groups of participants that the clinicians in this study found especially challenging to succeed with were participants who had an ongoing insurance case, were referred to occupational rehabilitation when they experienced themselves as not ready for work or wanting a disability pension and had a demanding private situation in addition to their health problems. The finding that it is challenging to work with participants that are not motivated for RTW is supported by other studies [33]. A lack of motivation was also identified as a strong risk factor for not returning to work. As Härkäpää and colleagues (2014) argue, there is a clear need to sort out and discuss what factors may hide behind weak contextual or situational motivation in the course of the occupational rehabilitation process [66]. In their view, this approach may alleviate uncertainty towards change, enhance their motivation to participate in occupational rehabilitation and re-think their future prospects of returning to work.

As another part of the interface with key stakeholders, the clinicians described interactive communication with their participants, other healthcare providers, case managers and employers to facilitate and co-ordinate the RTW process. Dialogue with workplaces, and in some instances, workplace visits to do workplace assessments, was seen as crucial for the RTW process by the clinicians. This allowed for the identification of possibilities for provisional or permanent work accommodations and the development of a RTW plan. As the clinicians in this study mentioned, and as identified in other research, such modifications might include reductions in physical and psychological job demands and modifications of work or work schedules on a temporary or permanent basis [35, 67].

In our study, the clinicians described interaction with different stakeholders, co-ordination of activities and follow-up of participants as challenging but essential. Among those who had an employer, fruitful dialogue and co-operation with representatives from the employer were regarded as decisive in helping their participants return to work. Among those who do not have an employer, but needed assistance to return to work, the clinicians described an increased need for co-operation with both employers and NAV consultants to help their participants back into the labour market. This multi-stakeholder interaction and integration of clinical and occupational intervention approaches in RTW are known to be one of the most challenging components in occupational rehabilitation. Researchers have pointed out the importance of differences in the beliefs and perceptions of different stakeholders [68, 69], different focuses of interest among stakeholders [70, 71] and insufficient co-ordination between employers and social security executives [72, 73].

In this study, we also found that the diagnosis and duration of sick leave were of low importance for the clinicians in their approaches and selection of interventions. Selecting interventions based on diagnoses alone is known to be difficult [74, 75]. As Glassel and colleagues (2011) suggest, a more comprehensive view including client demands, strategies and resources in daily life, the context around the individual and the social circumstances of their work situation must be taken into consideration [27]. Also, there is no unique and unified way to diagnose and select suitable interventions for persons with complex and subjective health complaints that characterise attendees in an occupational rehabilitation programme [3]. As shown in a large study among GPs in Scandinavia, choice of diagnosis varies greatly [76], and sick leave decisions are considered to be complex and individualised [77], not based on diagnosis [76]. Also, sick leave decisions are often a result of negotiations between the GP and their patient, influenced by conscious strategies from the GP to enhance RTW [78].

In our study, the clinicians pointed out the importance of revealing the participants’ thoughts of appropriate or inappropriate ways to behave when on sick leave. What participants think is inappropriate to do may be a hindrance in their recovery process. As the clinicians described, this could be related to self-imposed limitations in physical activity, contact with the workplace and other social contacts or activities in the public arena, often combined with changes in daily routines. This topic has been given little attention in occupational rehabilitation research. However, Jansson and Björklund (2007) found that long-term sick leave is connected to experiences of social stigma and a personal transition manifesting itself as a negative self-image, changes in life rhythms and restrictions in roles and activities [79].

An additional finding in our study is the clinicians’ extensive use of metaphors. The onion metaphor is well known, while statements such as *“grind down the threshold to working life”* seem to be more specific for occupational rehabilitation. It gives us an imaginary picture of a process where the clinicians try to slowly remove any obstacles that may be a hindrance for their participants’ RTW in different ways. Metaphors are typically used when no appropriate words are accessible, and can often transmit a whole story visually. As Ambrosini and Bowman describe, metaphors are means of capturing the continuous flow of experience; hence, they can be means of capturing tacit knowledge [80]. The use of metaphors is also one way for clinicians to provide understandable information for the participants, helping them to see analogies with their own situations [81].

The clinicians in our study described several success stories regarding their approaches to those on long-term sick leave, e.g., cases where their participants had been out of work for a long time or had a conflict with their supervisors. However, client-centred and interdisciplinary team-based approaches in occupational rehabilitation are not without challenges. All stakeholders involved, in addition to organisational, contextual and cultural factors influence whether they succeed. Good will, trust and co-operation are required to succeed with RTW arrangements [68, 70].

### Strength and limitations

This is the first study conducted in Norway that has collected information from a large group of occupational rehabilitation clinicians from different (in- and outpatient) clinics regarding the services they deliver. The results give valuable insight regarding how clinicians interact with participants and stakeholders to initiate changes that increases possibilities for RTW. The clinicians’ descriptions of the components of occupational rehabilitation programmes were in line with what are seen as core components in occupational rehabilitation [31]. Many studies in this field are criticised for providing little information about the content of the interventions and how they are delivered [45]. Our unique contribution is a more detailed description of some of the factors targeted by occupational rehabilitation clinicians by giving examples of what they do, say or provide to promote RTW among their participants. By recruiting clinicians registered for participation in a network meeting, we were able to conduct focus groups with many experienced clinicians from several inpatient and outpatient clinics in Norway who were working on interdisciplinary teams, which increases the transferability of the results. However, this study did not focus on variation in approaches between clinics. There are differences between clinics regarding how occupational rehabilitation programmes are organised and delivered, as well as which patient groups and professions are involved. There may also be differences in their theories and contextual factors that could influence their way of working. Nevertheless, the main results of the study were presented on an annual national network meeting for occupational rehabilitation clinicians in 2015. Several of those who participated in the focus groups in 2014 also took part in this meeting. The participants confirmed that the results provided an illustrative view of how they were working.

The moderators were from different professional backgrounds and the focus groups were carried out simultaneously, which might have influenced data collection through variations in follow-up questions and probing; moreover, this made follow-up of themes raised in one group with feedback from another impossible. Also, as researchers, we are not free from our theoretical and epistemological commitments [82]. Our different education and experiences influence our preconceptions and coding of data. However, all researchers wrote down their assumptions on how the clinicians were working with those on long-term sick leave before starting each focus group. These assumptions were discussed during the analysis process to prevent a biased interpretation of the results. We therefore considered our different backgrounds and knowledge in the field as a strength.

## Conclusion

Many of those admitted to occupational rehabilitation programmes in Norway have been on long-term sick leave close to one year or more, which makes the selection of interventions and the RTW process challenging for both clinicians and their participants. In order to facilitate RTW in this group, clinicians described a thorough, interdisciplinary mapping process as a crucial starting point. Also, to gain the trust of the participants, a client-centred approach characterised by sensitivity to the participants’ needs and emotional concerns was seen as a prerequisite to enable individual change processes. The clinicians described an interdisciplinary team approach where a variety of individual and/or group-based cognitive behavioural approaches, guided physical activities, education and dialogue with stakeholders were used to support participants in their RTW processes. These interventions were especially targeted at modifiable factors at the individual level, such as negative attitudes and beliefs, behaviour and abilities, including boundary setting in work and private life. The clinicians also approached obstacles for RTW in their participants’ surroundings depending on their individual circumstances and needs.

This study is expected to make a contribution to the research field by illuminating how occupational rehabilitation clinicians approach those on long-term sick leave by giving examples of what they do, say and provide to increase the participants’ abilities and possibilities to RTW.

## Abbreviations

GPs: General practitioners
ICF: International Classification of Functioning, Disability and Health
NAV: Norwegian Labour and Welfare Administration
RTW: Return to work
